# Supplementary material for: Bayesian refinement of protein structures and ensembles against SAXS data using molecular dynamics
Source: PLoS Comput Biol. 2017 Oct 18;13(10):e1005800. doi: 10.1371/journal.pcbi.1005800 (PMC5662244; doi:10.1371/journal.pcbi.1005800)
Supplement: S1 Archive — (BZ2) [file pcbi.1005800.s011.tar.bz2 › gromacs_4.62_bayesian_saxs_refi/share/html/online/speptide.html]

Getting started - Peptide


| Getting started - Peptide Main Table of Contents |

**VERSION 4.6  
Sat 19 Jan 2013**

---

## Contents

- Generating a topology file- Solvate the peptide- Generate an index file- Energy minimization- Molecular dynamics with position restraints- Unrestrained molecular dynamics- Analysis of trajectory files

## Ribonuclease S-peptide

Ribonuclease A is a digestive enzyme, secreted by the pancreas. The enzyme
can be cleaved by subtilisin at a single peptide bond to yield
Ribonuclease-S, a catalytically active complex of an S-peptide moiety
(residues 1-20) and an S-protein moiety (residues 21-124), bound together
by multiple non-covalent links (Stryer, 1988).

The S-Peptide has been studied in many ways, experimentally
as well as theoretically (simulation) because of the high a-helix
content in solution, which is remarkable in such a small peptide.

All the files of speptide are stored in the directory 
tutor/speptide. First go to this directory:cd speptide

To be able to simulate the S-Peptide we need a starting structure. This can
be taken from the protein data bank. There are a number of different
structure for Ribonuclease S, from one of which we have cut out the
first 20 residues, and stored it in
speptide.pdb.
Have a look at the file
  
  

|  |  |  |
| --- | --- | --- |
|  | more speptide.pdb   \| |

  

If you have access to a molecular
graphics program such as rasmol,
you can look at the molecule on screen, eg:
  
  

|  |  |  |
| --- | --- | --- |
|  | rasmol speptide.pdb   \| |

  

The following steps have to be taken to perform a simulation of the peptide.

1. Convert the pdb-file speptide.pdb
   to a GROMACS structure file and a GROMACS topology file.- Solvate the peptide in water- Perform an energy minimization of the peptide in solvent- Add ions if necessary (we will omit this step here)- Perform a short MD run with position restraints on the peptide- Perform full MD without restraints- Analysis

We will describe in detail how such a simulation can be done,
starting from a pdb-file.

### Generate a topology file (.top) from the pdb-file (.pdb)

Generate a molecular topology and a structure file in
format. This can be done with the pdb2gmx program:
  
  

|  |  |  |
| --- | --- | --- |
|  | pdb2gmx -f speptide.pdb -p speptide.top -o speptide.gro   \| |

  

Note that the correct file extension are added automatically to the
filenames on the command line.
You will only be asked to choose a forcefield, choose 0, but you can also
have pdb2gmx ask you
about protonation of residues, and about protonation of N- and C-terminus.
You can type
  
  

|  |  |  |
| --- | --- | --- |
|  | pdb2gmx -h   \| |

  

to see the available options.

The 
pdb2gmx program has generated a topology file
speptide.top and a
GROMACS structure file speptide.gro and it will
generate hydrogen
positions. The -p and -o options with he
filenames are optional; without them the files topol.top and 
conf.gro will be generated.
Now have a look at the output from pdb2gmx,
  
  

|  |  |  |
| --- | --- | --- |
|  | more speptide.gro   \| |

  

You will see a close resemblance to the pdb file, only the layout of
the file is a bit different.
Also do have a look at the topology
  
  

|  |  |  |
| --- | --- | --- |
|  | more speptide.top   \| |

  

You will see a large file containing the atom types, the physical
bonds between atoms, etcetera.

### Solvate the peptide in a periodic box filled with water

This is done using the programs
editconf and
genbox.
editconf
will make a rectangular box with empty space of user specified size
around the molecule.
genbox
will read the structure file and fill the box with water.
  
  

|  |  |  |
| --- | --- | --- |
|  | editconf -f speptide -o -d 0.5  genbox -cp out -cs -p speptide -o b4em   \| |

  

The program prints some lines of user information, like the volume of
the box and the number of water molecules added to your
peptide. genbox
also changes the topology file
speptide.top to include
these water molecules in the topology. This can been seen by looking
at the bottom of the
speptide.top file
  
  

|  |  |  |
| --- | --- | --- |
|  | tail speptide.top   \| |

  

You will see some lines like

```
[ system ]
; Name		Number
Protein		1
SOL		N
```

where N is the number of water molecules added to your system by
genbox.

It is also possible to solvate a peptide in another solvent such as
dimethylsulfoxide (DMSO), as has been done by
Mierke & Kessler, 1991.

### Generate index file (.ndx extension)

By default, most GROMACS programs generate a set of index groups to select
the most common subsets of atoms from your system (e.g. Protein, Backbone,
C-alpha's, Solute, etc.).
For the special cases when you need to select other groups than the
default ones, an index file
can be generated using make\_ndx.
This is an interactive program that lets you manipulate molecules,
residues and atom. It's use should be self-explanatory. To invoke the
program you would
  
  

|  |  |  |
| --- | --- | --- |
|  | make\_ndx -f b4em   \| |

  

but don't bother for now.

### Perform an energy minimization of the peptide in solvent

Now we have to perform an *energy minimization* of the
structure to remove the local strain in the peptide (due to generation
of hydrogen positions) and to remove bad Van der Waals contacts
(particles that are too close). This can be done with the
mdrun program which
is the MD and EM program. Before we can use the
 mdrun program
however, we have to preprocess the topology file (
speptide.top), the
structure file (
speptide.gro) and a
special parameter file (em.mdp). Check
the contents of this file
  
  

|  |  |  |
| --- | --- | --- |
|  | more em.mdp   \| |

  

Preprocessing is done with the preprocessor called
grompp. This reads
up the files just mentioned:
  
  

|  |  |  |
| --- | --- | --- |
|  | grompp -v -f em -c b4em -o em -p speptide   \| |

  

In this command the -v option turns on verbose mode, which
gives a little bit of clarifying info on what the program is doing.
We now have made a *run input file* (em.tpr) which
serves as input for the
mdrun program. Now
we can do the energy minimization:
  
  

|  |  |  |
| --- | --- | --- |
|  | mdrun -v -s em -o em -c after\_em -g emlog   \| |

  

In this command the -v option turns on verbose mode again.
The -o option sets the filename for the trajectory file,
which is not very important in energy minimizations. The -c
option sets the filename of the structure file after energy
minimization. This file we will subsequently use as input for the MD
run. The energy minimization takes some time, the amount depending on
the CPU in your computer, the load of your computer, etc. The
mdrun program is
automatically *niced*; it runs at low priority. All programs
that do extensive computations are automatically run at low
priority. For most modern workstations this computation should be a
matter of minutes. The minimization is finished when either the
minimization has converged or a fixed number of steps has been
performed. Since the system consists merely of water, a quick check
on the potential energy should reveal whether the minimization was
successful: the potential energy of 1 SPC water molecule at 300 K is
-42 kJ mole^-1^. Since we have about 2.55e+03
SPC molecules the potential energy should be about -1.1e+5 kJ
mol^-1^. If the potential energy after minimization is lower
than -1.1e+05 kJ mol^-1^ it is acceptable and the
structure can be used for MD calculations. After our EM calculation
the program prints something like:

```
STEEPEST DESCENTS converged to 2000
  Potential Energy  = -1.19482e+05
```

which means our criterium is met, and we can proceed to the next step.

### Perform a short MD run with position restraints on the peptide

Position restrained MD means Molecular Dynamics in which a part of the
system is not allowed to move far off their starting positions. To be
able to run with position restraints we must add a section to the
speptide.top file,
describing which atoms are to be restrained. Such a section is
actually generated by the
pdb2gmx program. In the
topology file it looks like

```
#ifdef POSRES  

#include "posres.itp"  

#endif
```

In the topology file we use
conditional inclusion, i.e. only if a variable POSRES is set
in the preprocessor do we include the file, this allows us to use the
same topology file for runs with and without position restraints. In
the pr.mdp parameter file
for the position restraints this variable is set indeed:

```
define              =  -DPOSRES
```

At last we can generate the input for the position restrained mdrun:
  
  

|  |  |  |
| --- | --- | --- |
|  | grompp -f pr -o pr -c after\_em -r after\_em -p speptide   \| |

  

Now it's MDrun time:
  
  

|  |  |  |
| --- | --- | --- |
|  | mdrun -v -s pr -e pr -o pr -c after\_pr -g prlog >& pr.job &   \| |

  

This run is started in the background (it will take a while), you
can watch how long it will take by typing:
  
  

|  |  |  |
| --- | --- | --- |
|  | tail -f pr.job   \| |

  

With the Ctrl-C key you can kill the tail command.
A good check of your simulation is to see whether density and potential
energies have converged:
  
  

|  |  |  |
| --- | --- | --- |
|  | g\_energy -f pr -o out -w   \| |

  

The 
g\_energy program will prompt you to select a number of energy terms
from a list. For potential energy type:
  
  

|  |  |  |
| --- | --- | --- |
|  | 9 0   \| |

  

If you have the xmgr program installed it will automatically pop up on your
screen with the energy plot. You can do the same for the density
and other energy terms, such as Solvent-Protein interactions.

### Perform full MD without restraints

Full MD is very similar to the restrained MD as far as GROMACS is
concerned. Check out the full.mdp for details.
  
  

|  |  |  |
| --- | --- | --- |
|  | grompp -v -f full -o full -c after\_pr -p speptide   \| |

  

Then we can start mdrunning
  
  

|  |  |  |
| --- | --- | --- |
|  | mdrun -v -s full -e full -o full -c after\_full -g flog >& full.job &   \| |

  

You should do similar convergence checks (and more!) as for the position
restrained simulation.
  

---

  

### Analysis

We will not describe analysis in detail, because most analysis tools
are described in the Analysis chapter of the printed manual.
We just list a few of the possibilities within GROMACS. By now you should be
able to start programs yourself.

1. View the trajectory on your own X-screen (program
   ngmx).
     
     

   |  |  |  |
   | --- | --- | --- |
   |  | ngmx -s pr -f full   \| |

     
   What happens to the peptide?
2. The Root Mean Square Deviation (RMSD) with respect to the crystal
   structure (program
   g\_rms) is a measure of how well the
   crystal (starting) structure is maintained in the simulation.
   The RMSD at time t is computed as
   <(**r**(t)-**r**(0))^2^>^1/2^
   after performing a least squares super position of the structure at
   time t onto the structure at time 0.
     
     

   |  |  |  |
   | --- | --- | --- |
   |  | g\_rms -s pr -f full -o rmsd   \| |

     
   Select the 1 for the number of groups, and select Calpha (Ca) for fitting
   and for computing the RMSD. View the output graph with xmgrace.
     
     

   |  |  |  |
   | --- | --- | --- |
   |  | xmgrace rmsd.xvg   \| |

     
   Does the RMSD
   converge within the simulation? If not, what does this indicate?

   - The Radius of Gyration (Rg, program
     g\_gyrate)) is a measure of the size of the
     protein. It is computed as the mean square distance of atoms from the center
     of mass of the molecule: Rg(t) = SUM_i=1 .. N_
     (**r**_i_-**r**_com_)^2^
       
       

     |  |  |  |
     | --- | --- | --- |
     |  | g\_gyrate -s pr -f full -o gyrate   \| |

       
     View the graph with xmgrace:
       
       

     |  |  |  |
     | --- | --- | --- |
     |  | xmgrace gyrate.xvg   \| |

       
     Does the radius of gyration change during the
     simulation?

     - The Ramachandran Plot shows whether the backbone torsion angles
       (φ/ψ) of your
       peptide are within the allowed region.
       (program g\_rama).
         
         

       |  |  |  |
       | --- | --- | --- |
       |  | g\_rama -s pr -f full -o rama   \| |

         
       View the graph with xmgrace:
         
         

       |  |  |  |
       | --- | --- | --- |
       |  | xmgrace rama.xvg   \| |

         
       Here you have all the backbone torsion angles from the trajectory.
       Are all the angles in the allowed region?
       What kind of structure do the angles indicate?

       - A salt bridge analysis (program
         g\_saltbr) will tell you whether
         there are any saltbridges formed or broken during the simulation.
         It will also tell you about repulsive electrostatic interactions.
           
           

         |  |  |  |
         | --- | --- | --- |
         |  | g\_saltbr -s pr -f full -t 0.5 -sep   \| |

           
         This will generate four graphs (in xmgrace format). Two of them refer to
         attractive interactions.
         You can view the graphs with xmgrace:
           
           

         |  |  |  |
         | --- | --- | --- |
         |  | xmgrace sb-GLU2:ARG10.xvg sb-GLU2:LYSH7.xvg -legend load   \| |

           
         Which interaction is the strongest?
         Look at the peptide molecule again (using rasmol).
         Which atoms form the closest contact in the saltbridge?

         - Secondary Structure analysis (program
           my\_dssp).
           This analysis uses the dssp (dictionary of secondary structure in proteins,
           Kabsch & Sander, 1983) software.
             
             

           |  |  |  |
           | --- | --- | --- |
           |  | my\_dssp -s pr -f full   \| |

             
           Select protein when asked to select a group.
           You can postprecess the output file with:
             
             

           |  |  |  |
           | --- | --- | --- |
           |  | xpm2ps -f ss.xpm -o ss.eps   \| |

             
           This will give you a postscript file which you can either print or
           view with xpsview.
             
             

           |  |  |  |
           | --- | --- | --- |
           |  | xpsview ss.eps   \| |

             
           What happens to the Alpha helix (in blue)?


  

---

  

You have been witness of a full MD simulation starting from a pdb-file.
It's that easy, but then again, maybe it was not that easy. The
example presented here is a *real* example, this is how a
production run should be performed, the complexity is in the process
itself and not in the software (at least, that's our opinion).

---


### Go to the next step


---
